# The Influence of Different Inter-Trial Intervals on the Quantification of Intracortical Facilitation in the Primary Motor Cortex

**DOI:** 10.3390/bioengineering10111278

**Published:** 2023-11-02

**Authors:** Milan Pantovic, Rhett Boss, Kevin J. Noorda, Mario I. Premyanov, Daniel G. Aynlender, Erik W. Wilkins, Sage Boss, Zachary A. Riley, Brach Poston

**Affiliations:** 1 Health and Human Performance Department, Utah Tech University, St. George, UT 84770, USA; milan.pantovic@utahtech.edu; 2 School of Medicine, University of Nevada-Las Vegas, Las Vegas, NV 89154, USA; bossr1@unlv.nevada.edu (R.B.); noordk1@unlv.nevada.edu (K.J.N.); premayno@unlv.nevada.edu (M.I.P.); aynlende@unlv.nevada.edu (D.G.A.); 3 Department of Kinesiology and Nutrition Sciences, University of Nevada-Las Vegas, Las Vegas, NV 89154, USA; wilkie1@unlv.nevada.edu; 4 School of Life Sciences, University of Nevada-Las Vegas, Las Vegas, NV 89154, USA; bosss1@unlv.nevada.edu; 5 Department of Kinesiology, Indiana University Purdue University Indianapolis, Indianapolis, IN 46202, USA; zariley@iupui.edu

**Keywords:** transcranial magnetic stimulation, motor evoked potential, intracortical facilitation, short-interval intracortical inhibition, electromyography

## Abstract

Intracortical facilitation (ICF) is a paired-pulse transcranial magnetic stimulation (TMS) measurement used to quantify interneuron activity in the primary motor cortex (M1) in healthy populations and motor disorders. Due to the prevalence of the technique, most of the stimulation parameters to optimize ICF quantification have been established. However, the underappreciated methodological issue of the time between ICF trials (inter-trial interval; ITI) has been unstandardized, and different ITIs have never been compared in a paired-pulse TMS study. This is important because single-pulse TMS studies have found motor evoked potential (MEP) amplitude reductions over time during TMS trial blocks for short, but not long ITIs. The primary purpose was to determine the influence of different ITIs on the measurement of ICF. Twenty adults completed one experimental session that involved 4 separate ICF trial blocks with each utilizing a different ITI (4, 6, 8, and 10 s). Two-way ANOVAs indicated no significant ITI main effects for test MEP amplitudes, condition-test MEP amplitudes, and therefore ICF. Accordingly, all ITIs studied provided nearly identical ICF values when averaged over entire trial blocks. Therefore, it is recommended that ITIs of 4–6 s be utilized for ICF quantification to optimize participant comfort and experiment time efficiency.

## 1. Introduction

Transcranial magnetic stimulation (TMS) is a non-invasive technique that allows the assessment of corticospinal excitability at rest and during muscle activation [1,2,3,4]. Accordingly, a single suprathreshold TMS pulse applied to the primary motor cortex (M1) elicits an electromyographic (EMG) response termed the motor evoked potential (MEP), which is used as an index of corticospinal excitability [5]. In addition, a test MEP can be paired with a subthreshold conditioning TMS pulse (paired-pulse TMS) in condition-test paradigms at various inter-stimulus intervals (ISIs) and stimulation intensities to measure several inhibitory and excitatory intracortical interneuronal pathways in M1 [6,7,8]. Short-interval intracortical inhibition (SICI) and intracortical facilitation (ICF) are the most studied and described of these pathways [6,9,10,11].

ICF is examined by applying a subthreshold conditioning TMS pulse briefly followed by a suprathreshold TMS test pulse at ISIs of 6–25 milliseconds with 10 milliseconds being the most common. This leads to facilitation of the condition-test MEP amplitudes compared to single-pulse test MEP amplitudes that are randomly intermixed in the same block of TMS testing. Although findings have been somewhat mixed, the balance of evidence suggests that ICF is most likely mediated by populations of intracortical interneurons in M1 and not due to subcortical or spinal mechanisms [6,10,12,13]. Accordingly, ICF is thought to be mainly mediated by excitatory glutamatergic neurons in M1 and has been shown to be altered (either increased or decreased) in several motor disorders [6,10,13]. For this reason, along with the fact that ICF is often quantified with several other measures of intracortical inhibition and facilitation, accurate quantification of ICF is important in the study of movement control.

Due to the significance and prevalence of single and paired-pulse TMS techniques, extensive research has endeavored to determine optimal methodological procedures [14] and consensus guidelines for TMS studies involving these methods [10]. For example, an international panel of 42 experts identified 21 methodological items that should be reported or controlled in TMS studies [15]. One of the items was the time between MEP trials (hereafter termed inter-trial interval; ITI) and 82% of the expert panel respondents reported that this item was important or very important to control, whereas 87% reported that ITI should be reported always or most of the time [15]. Despite these recommendations and the observations that ITI can influence other evoked responses such as the Hoffman reflex [16,17] and auditory evoked potentials [18], there currently appears to be no consensus guidelines and little research available regarding the appropriate ITI to utilize between single-pulse MEPs, but especially paired-pulse MEPs. Thus, ITI has been underemphasized and underappreciated relative to many of the other stimulation parameters that are involved in paired-pulse TMS such as stimulation intensities and ISIs to name a few. Accordingly, both a wide range of ITIs and several methods of varying the ITI on a trial-to-trial basis have been used in the literature on single-pulse TMS. For instance, protocols such as “an interval of 7 s (10% variance) [19]”, “less than every 5 s [11]”, “the intervals between the stimuli usually ranged between 20 and 30 s [20]”, “pseudo randomly at intervals ranging between 3.5 and 7 s [21]”, “every 15 s [22]”, “varied between 1.5 and 2.5 s [23]”, and many others have been used. Furthermore, observation suggests that the most common ITIs used in the literature are between 4 and 6 s, although the vast majority of TMS studies do not even report the ITI at all.

It is difficult to determine why ITI has received considerably less attention compared to other TMS parameters of stimulation. Perhaps ITI has simply been overlooked or this is due to the widespread view that only repetitive TMS techniques involving high stimulation rates and extended stimulation periods lead to effects that persist for a significant time after the stimulation [24,25,26]. Accordingly, the commonly held belief that each MEP is an independent event [27] was supported by the observation that a single TMS pulse given to M1 increased cortico-muscular coherence for only 300–800 milliseconds before values returned to baseline [28]. However, a few single-pulse TMS studies do exist that have shown that the amplitudes of successive MEPs evoked in a block of trials may not be as time-invariant as commonly thought [29]. For example, one extensive study found that single-pulse MEP amplitudes elicited at a constant stimulation intensity were significantly lower at short ITIs (1, 2, 3, and 5 s) compared with a long ITI of 10 s. This effect was most prominent in the first 1–10 MEP trials of a 30 MEP trial block. Furthermore, MEP recruitment curves obtained at rest using both increasing and decreasing TMS stimulation intensities exhibited hysteresis when the average ITI was 5 s, but not when the average ITI was 20 s [27]. Although recruitment curve construction involves the usage of a wide range of stimulation intensities versus the constant stimulation intensities used in other types of single and paired-pulse TMS measures, these findings lend some further support to the idea that MEP measurements may be influenced by ITI. Accordingly, another single-pulse TMS study reported similar results as a 5 second ITI resulted in lower average MEP amplitudes in a block of 25 MEP trials compared to ITIs of 10, 15, and 20 s [30].

However, no studies have examined the influence of different ITIs on paired-pulse TMS measurements. Accordingly, the ICF values attained during a typical block of TMS trials involving intermingled condition-test trials and test MEP trials using different ITIs have never been investigated. This is surprising given the aforementioned single-pulse ITI studies and the fact that single-TMS pulses serve as the test MEP in paired-pulse TMS paradigms as well as the test MEP being a component of the condition-test MEP. Taken together, these lines of reasoning raise the possibility that different ITIs, including those that have been used in many paired-pulse TMS studies, could have resulted in skewed, different, or even erroneous ICF values. Therefore, the primary purpose was to examine the influence of different ITIs on measurements of ICF at rest. This was accomplished by quantifying ICF during 4 blocks of trials at ITIs of 4, 6, 8, and 10 s. Based on the limited number of single-pulse TMS studies [27,29], it was hypothesized that ICF amplitude would differ for the short (4 s) compared with long (6, 8, and 10 s) ITI blocks. Furthermore, it was predicted that this would be due to an initial suppression (lower facilitation) in the first few trials and not a serial reduction in ICF amplitude over the entire trial block. The secondary purpose was to examine the influence of different ITIs on measurements of single-pulse MEPs at rest. This was accomplished by quantifying single-pulse MEPs at rest during 2 blocks of trials at ITIs of 4 and 10 s. These separate single-pulse blocks had the dual purpose of serving as control blocks for the ICF blocks and as comparisons to previous TMS studies that assessed the effect of ITI on single-pulse MEP amplitudes [27,29,30]. Based on these previous studies, it was hypothesized that MEP amplitude would be lower for the 4-second ITI block compared to the 10-second ITI block. Finally, it was expected that this would be due to an initial MEP suppression in the first few trials of the 4-second ITI block and not a serial reduction in MEP amplitude over the entire trial block.

## 2. Materials and Methods

### 2.1. Participants

Twenty young adults participated in the study (10 males and 10 females; mean age: 25.0 ± 2.3). All participants were determined to be right-handed based on the Edinburgh Handedness Inventory [31]. Participants reported that they were free of any psychiatric or neurological disorders and had no uncontrolled medical conditions. In addition, none of the participants met the exclusion criteria for non-invasive brain stimulation studies [14]. Finally, all experimental procedures were conducted in accord with the Declaration of Helsinki and the Institutional Review Board at the University of Nevada, Las Vegas approved the study (protocol number: 1445199).

### 2.2. Experimental Design

**Figure 1:**
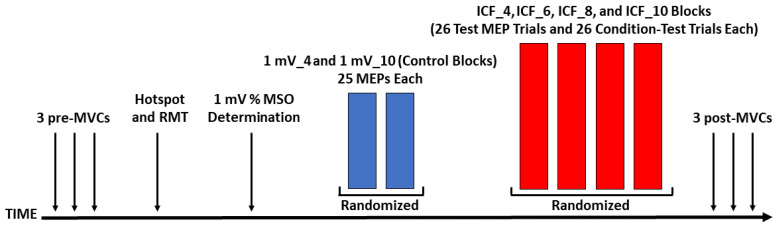
Schematic of the experimental protocol. Each experiment comprised 7 steps that included: 3 MVCs (pre), motor hotspot localization, RMT quantification, 1 mV stimulation intensity determination, the 1 mV_4 and 1 mV_10 control blocks, the ICF_4, ICF_6, ICF_8, and ICF_10 blocks, and 3 MVCs (post).

The study utilized a within-subjects experimental design and all participants completed one experimental session that lasted about 2 h. The size of the sample of participants (*n* = 20) was based on the available previous studies on ITI that involved single-pulse TMS [27,29,30,32,33,34]. Collectively, these studies had a sample size range of 8–17; participants and an average sample size of 12.5. Thus, it was decided that 20 participants should be more than enough to demonstrate the influence of ITI on ICF if this phenomenon were to exist. Each experimental session proceeded in the following set of 7 steps: (1) baseline maximum voluntary contraction (pre-MVC) measurements; (2) motor hotspot localization; (3) resting motor threshold quantification; (4) determination of the stimulation intensity as a percentage of maximum stimulator output (% MSO) required to elicit a MEP of approximately 1 mV with a 10-second ITI; (5); 2 control blocks that involved single-pulse MEPs evoked with either a 4-second ITI or a 10-second ITI (hereafter referred to as the 1 mV_4 and 1 mV_10 conditions); (6) 4 separate trial blocks that involved paired-pulse TMS measurement of ICF at 4 ITIs of 4, 6, 8, and 10 s (hereafter referred to as ICF_4, ICF_6, ICF_8, and ICF_10 conditions), and (7) post-MVCs. Figure 1 depicts the major experimental steps of the experimental protocol while the methodological details of each step are provided below in subsequent sections.

### 2.3. Experimental Arrangement

The experimental arrangement (equipment setup and participant posture) was similar to previous studies [35,36]. Briefly, participants were seated comfortably in a chair next to a small table such that their shoulder was abducted to about 45 degrees, the elbow was flexed to approximately 90 degrees, the forearm rested on a flat block that was placed on the table, and the wrist was placed in a neutral position with the hand prone. Most importantly, participants were required to keep this same posture during all TMS testing as changes in upper limb and shoulder posture can influence MEPs in hand muscles [37,38]. A computer monitor was located at eye level about a meter in front of the participants to provide feedback of the EMG activity of the right FDI muscle, which served as the target muscle for all TMS and experimental testing. Participants were given detailed and strict instructions on how to use this visual feedback to ensure that the FDI muscle remained at rest during all of the TMS recordings. In addition, this computer screen was also constantly monitored by one of the investigators to further ensure that participants were keeping the FDI at rest throughout the TMS testing blocks.

TMS was performed using two Magstim 200^2^ stimulators connected by a Bistim module and delivered through a standard double 70 mm remote control figure-of-eight coil. All single-pulse and paired-pulse TMS measurements were completed with the TMS unit in Bistim Mode [39]. The TMS coil was held in such a way that it was kept tangential to the scalp with the handle orientated backward and laterally at a 45-degree angle relative to the midline. An investigator placed the TMS coil over the location corresponding to the “motor hot spot” of the FDI muscle of the left M1 to evoke MEPs in the FDI of the right hand [40]. The EMG activity of the FDI muscle was recorded with prewired disposable surface electrodes arranged in a belly tendon montage. All EMG signals were acquired using Cambridge Electronic Design (CED; Cambridge, UK) hardware (1902 amplifiers, micro 1401 data acquisition interface) and software (Signal 5.04).

### 2.4. Experimental Procedures

#### 2.4.1. MVCs

MVCs were conducted using standard methodology and similar to previous studies [35,41,42]. Since the FDI was the muscle of interest, MVCs were performed using index finger abduction as almost all of the index finger abduction force is produced by the FDI. A manipulandum instrumented with a force transducer was situated on the table very near the end of the block where their hand was placed. Thus, participants could exert force on the force transducer at the level of the proximal interphalangeal joint with the index finger. For all MVC trials, participants were instructed to generate their maximum force in the shortest time possible and to hold this maximum for ~5 s [42]. Visual feedback of the FDI abduction force was given in the form of a red force trace that scrolled across the computer monitor. Three MVC trials were completed at the beginning (pre-MVCs) and at the end of the experimental session (post-MVCs) immediately after the ICF blocks. A rest period of one minute was enforced between all MVC trials.

The rationale for performing MVCs was to provide some confirmation that the ability to voluntarily activate the FDI muscle and by extension factors such as alertness or arousal [5] that can influence MEPs had not substantially declined over the course of the experiment due to central fatigue. Although the chances of significant central fatigue in the current study were likely extremely remote as all procedures were obtained at rest, the concentration required to complete the experiment could theoretically lead to mental fatigue. It has been known for a long time that mental fatigue even in the absence of physical exercise can reduce voluntary activation [43] and therefore potentially even resting MEP measurements in a long experimental session.

#### 2.4.2. Motor Hotspot Localization

Suprathreshold TMS pulses were delivered as the coil position was optimized so that the point on the scalp where the largest MEPs could be evoked. This location was denoted as the FDI motor hot spot, the coil position was marked on a scalp cap, and the scalp cap position on the head was outlined with a mark on the forehead [40]. Accordingly, all MEPs in the experiment were evoked using this location.

#### 2.4.3. RMT

The RMT was measured for each participant and was defined as the lowest stimulation intensity required to induce a 50-microvolt peak-to-peak MEP in at least 5 out of 10 consecutive TMS trials. Subsequently, the RMT value was used to determine the stimulation intensity for the conditioning pulses of the ICF measurements for each participant.

#### 2.4.4. mV Stimulation Intensity (% MSO) Determination

Suprathreshold TMS pulses were applied and the stimulation intensity was adjusted while MEPs were monitored and quantified online until the MEPs evoked were as close as possible on average to a 1 mV peak-to-peak amplitude [44]. This initial testing was completed to determine the stimulation intensity as a percentage of maximum stimulator output (% MSO) to elicit an average MEP of approximately 1 mV. This stimulation intensity was then used for all subsequent experimental blocks (the single-pulse MEPs in the control blocks and all the test MEPs in the ICF blocks). This testing was completed using an ITI of 10 s so as to get the 1 mV MEP stimulation intensity at the longest ITI used in the study. This was based on previous studies [27,29,30] that this should be the true 1 mV MEP value as an ITI of 10 s should not be influenced by time-dependent effects. Most importantly, great attention and time were devoted to identifying the % MSO value that elicited MEPs as close to 1 mV as possible. Briefly, this was completed by monitoring and estimating the MEP amplitudes online using a software script in Signal and resetting the program as needed and changing the stimulation intensity until the investigators were confident the best possible value had been identified. Finally, this value was used for all of the subsequent single-pulse and ICF trial blocks that were used for analysis.

#### 2.4.5. Control Blocks

Two separate control blocks using ITIs of 4 and 10 s (hereafter referred to as the 1 mV_4 and 1 mV_10) consisting only of single-pulse MEPs were performed in randomized order (Figure 1). These blocks were included to serve the dual purpose of controls for the single-pulse test MEPs elicited in the ICF blocks and to compare the results to previous ITI studies that involved only single-pulse TMS [27,29,30]. Accordingly, only the shortest and longest ITIs of 4 and 10 s used in the subsequent ICF blocks were investigated in the control blocks. This was decided upon because any ITI differences were most likely to be observed between the shortest and longest ITIs and in the interest of time as it was already a long experiment with the primary focus being on the ICF blocks. A total of 25 MEPs was collected in both blocks using the 1 mV stimulation intensity (% MSO) for each participant that was established in the previous step. This stimulation intensity to evoke an MEP of 1 mV was used in the control blocks as it is the typical number used to measure MEP changes before and after numerous types of interventions (e.g., transcranial direct current stimulation, exercise, various behavioral state changes, etc) in TMS studies. Most importantly, the 1 mV MEP is almost always used as the test pulse stimulation intensity in paired-pulse TMS. The number of 25 MEP trials per block was selected for the following interrelated reasons: (1) the most comprehensive study on the topic [45] found that 20–30 MEPs generally provide the best trade-off between the minimum number of trials to provide valid results for average MEP amplitude of a block of trials at both the individual and group level; (2) this is also the range of MEPs that the same authors determined is broadly applicable and practical to accomplish due to time and other constraints inherent in most TMS studies; and (3) the two most relevant prior single-pulse TMS studies involving ITI used 25 and 30 trials per block [29,30].

#### 2.4.6. ICF Blocks

ICF was quantified in 4 separate trial blocks using ITIs of 4, 6, 8, and 10 s (hereafter referred to as ICF_4, ICF_6, ICF_8, and ICF_10) with the blocks performed in a randomized order (Figure 1). The ITI employed was the only difference across the four ICF trial blocks. Accordingly, the ICF protocol was always administered using the same coil to deliver the subthreshold conditioning pulse followed by a suprathreshold test pulse separated by an interstimulus interval (ISI) of 10 milliseconds. The conditioning pulse stimulation intensity was set to 90% of RMT, whereas the test pulse stimulation intensity was set to the previously determined 1 mV stimulation intensity as is almost always completed in paired-pulse TMS studies. This combination of ICF parameter values for ISI, conditioning pulse intensity, and test pulse intensity were chosen as they are generally the most common in the literature and found to be the most optimal for observing ICF in the most systematic study on the topic [11].

All ICF blocks involved a total of 52 TMS trials with 26 trials involving single-pulse TMS (test MEPs alone) and 26 trials involving paired-pulse TMS (condition-test MEPs) that collectively were used to calculate ICF. Within each ICF trial block, these two types of trials (termed “states” in Signal software) were presented in a semi-randomized order, which in this case means that every two TMS trials were randomized between the trial types (states). The number of 26 trials per each type of trial was selected for reasons similar to those mentioned for the 25 MEP number in the previous section. In addition, this allowed at least 25 trials for each trial type in the event that the first 2 trials would have to be discarded (see Section 4.4 of the Discussion), although this turned out to not be the case. Accordingly, each ICF block consisted of 26 test MEP trials, 26 condition-test MEP trials, and therefore 26 ICF measurements. Accordingly, ICF was calculated as the condition-test MEP amplitude divided by the test MEP amplitude and expressed as a percentage.

The 4 ITIs used for investigation were selected for the following interrelated reasons: (1) although some previous single-pulse TMS studies examined ITIs below 4 s [29,33], it was determined in extensive pilot testing that any ITI below 4 s for ICF testing was not practical. This was because the TMS device could easily skip trials due to the capacitors not charging quickly enough if a participant required relatively high stimulation intensities for the test and/or condition-test MEPs. Relatedly, ITIs shorter than 4 also were found to be prone to coil overheating in a small number of instances during piloting. Obviously, any skipped trials would render the results of this particular study uninterpretable; (2) an ITI of 4 s should still have been able to detect time-varying changes in MEP amplitudes due to ITI if they were present, based on previous single-pulse studies [27,29,30]. Similarly, an ITI of 10 should be adequate to reveal valid MEP amplitude according to the same studies; (3) ITIs over 10 are essentially impractical for the vast majority of TMS experiments as these conditions lead to experiment times that are too long and uncomfortable for both the experimenter and the participant. This was readily apparent in pilot testing and would also reduce the total number of TMS blocks or conditions that could be completed in typical TMS experiments.

### 2.5. Data Analysis

All MEP and MVC data were analyzed using customized scripts written in Signal 5.04 software (Cambridge Electronic Design, Cambridge, UK). The investigators who performed the data collection experiments did not participate in the data reduction or data analysis aspects of the study [46].

#### 2.5.1. MVC Force, MVC EMG, RMT, and 1 mV (% MSO) Stimulation Intensity Analyses

MVC force was calculated as the average force produced over the 3–5 s plateau period for each trial and the highest MVC among each set of pre and post-MVCs was used for analysis [36]. The maximum MVC EMG was calculated over this same time period and the highest MVC EMG among each set of pre and post-MVCs were used for analysis. MEP size was always calculated as the peak-to-peak amplitude for each individual MEP in all the analyses below. The RMT and 1 mV stimulation intensity (% MSO) are reported as the group averages to provide information on the participant characteristics and the numbers used to determine the ICF test and condition pulse stimulation intensities.

#### 2.5.2. Control Block Analyses

**Figure 2:**
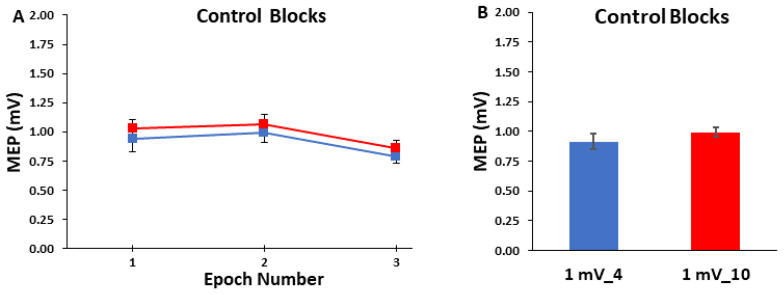
MEP amplitude in the control blocks. (**A**) The MEP amplitude as a function of Epoch number. MEP amplitude was similar for the 1 mV_4 condition and 1 mV_10 conditions and across the three epochs; (**B**) The MEP amplitude was similar for the 1 mV_4 and 1 mV_10 control blocks when averaged over the whole block.

**Figure 3:**
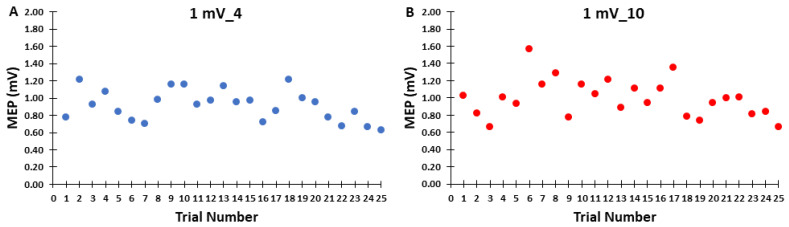
MEP amplitude as a function of trial number for the control blocks. Each point represents the average of all twenty participants for a given trial in each of the two blocks. (**A**) MEP amplitude as a function of trial number for the 1 mV_4 condition; (**B**) MEP amplitude as a function of trial number for the 1 mV_10 condition.

The MEP amplitudes in the control blocks were averaged in three different ways for analysis and illustration purposes. First, possible changes in MEP amplitude over the time course of the control blocks were analyzed by dividing the 25 MEP trials in each block into 3 separate time epochs of consecutive MEP trials (Epoch 1: trials 1–8; Epoch 2: trials 9–16; and Epoch 3 trials 17–25; Figure 2A). Thus, Epochs 1 and 2 consisted of 8 trials, whereas Epoch 3 consisted of 9 trials. This was similar to a previous single-pulse study that divided 30 MEP trial blocks into 3 sub-blocks of 10 for analysis [29]. The reason for Epochs 1 and 2 ultimately consisting of 8 trials instead of 9 such as Epoch 3 (or some similar arrangement of exactly equal trials per epoch) was in the event that the first trial of each block would have to be discarded (see Section 4.4 of the Discussion), although this turned out to not be the case. Second, MEP amplitude was also quantified as the average of all 25 MEP trials in each control block (Figure 2B). This was mainly completed to illustrate the overall average MEP amplitude for the control blocks as the same information is contained in the prior step and associated figure, but the overall averages are not as easy to ascertain. Third, to further evaluate possible changes in MEP amplitude over the time course of the control blocks, the average MEP amplitudes of all 20 participants were quantified for each of the 25 trials and plotted. This was completed primarily for visual assessment and illustrative purposes of the series of 25 trials in each control block (Figure 3).

#### 2.5.3. ICF Block Analyses

**Figure 4:**
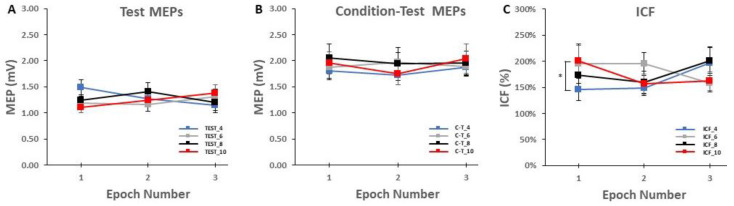
Test MEPs, condition-test MEPs, and ICF values in the ICF blocks. (**A**) The MEP amplitude as a function of Epoch number for the test MEP trials only. MEP amplitude was similar for the four test MEP ITI conditions across the three epochs; (**B**) The MEP amplitude as a function of Epoch number for the condition-test MEP trials only. MEP amplitude was similar for the four condition-test ITI conditions across the three epochs; (**C**) ICF values as a function of Epoch number. ICF was lower for the ICF_4 condition compared with the ICF_10 condition, but only in the first epoch (*p* = 0.015). All other ICF values were similar for the four ICF ITI conditions across the 3 epochs. * indicates the significant pairwise comparison between ICF_4 and ICF_10 in Epoch 1.

**Figure 5:**
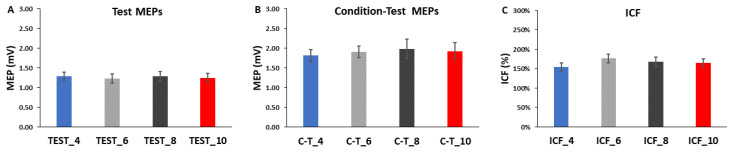
The overall block averages for test MEPs, condition-test MEPs, and ICF values in the ICF blocks. (**A**) There were no differences in average MEP values for the test MEPs for any of the ITIs. (**B**) There were no differences in average MEP values for the condition-test MEPs for any of the ITIs. (**C**) Thus, there was no difference in ICF for the four ITIs when comparing the overall block averages.

**Figure 6:**
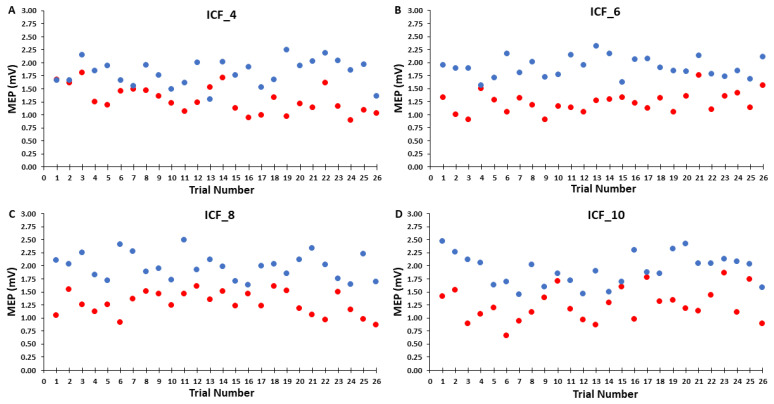
Test MEP and condition-test MEP amplitudes as a function of trial number for the ICF blocks for the four ITIs are depicted for illustration (**A**–**D**). Test MEP trials are indicated in red and condition-test MEP trials are indicated in blue. Each data point represents the average MEP amplitudes of all 20 subjects for a given trial.

Similar to the control blocks, the MEP amplitudes in the ICF blocks were processed in three different ways for analysis and illustration purposes. First, groups of MEP trials were also divided into epochs with the exception that the corresponding Epochs 1, 2, and 3 consisted of 16, 16, and 18 trials, respectively. In addition to the obvious reasons for these trial blocks involving paired-pulse TMS and more total trials, the major reason for the slightly different number of trials per epoch was similar to the previous explanation regarding the possibility of discarding the first two trials, which also that did not materialize (see Section 4.4 of the Discussion). Accordingly, the average MEP amplitudes of the test MEP trials, condition-test MEP trials, and therefore ICF calculation were quantified as the average 8, 8, and 9 trials for each of these measures in the ICF blocks and used for analysis (Figure 4A–C). Thus, ICF was calculated as the condition-test MEP amplitude divided by the test MEP amplitude and expressed as a percentage according to standard practice [6,9,10,11]. Second, the average MEP amplitudes for each of the 26 test MEP trials, 26 condition-test MEP trials, and therefore 26 ICF measurements were taken for analysis and plotted (Figure 5). This was mainly completed to illustrate the overall average MEP amplitude for these measures as the same information is contained in the prior step and associated figure, but the overall averages are not as easy to visualize. Third, to further visualize changes in test and condition-test MEP trials individually over the time course of the ICF blocks, the average test MEP and condition-test MEP amplitudes of all 20 participants were quantified for each of their respective 26 trials and plotted. This was compelted primarily for visual assessment and illustrative purposes This was completed primarily for visual assessment and illustrative purposes of the series of trials that comprised each ICF block (Figure 6).

### 2.6. Statistical Analysis

#### 2.6.1. MVCs

The pre-MVC and post-MVC conditions were compared with a paired *t*-test. Similarly, the pre-MVC EMG and post-MVC EMG were also compared with a paired *t*-test.

#### 2.6.2. Control Blocks

To analyze possible differences in MEP amplitude over the time course of the control blocks a 2 *Control Block* (1 mV_4, 1 mV_10) × 3 *Epoch* (1, 2, 3) within-subjects ANOVA was utilized. Post hoc comparisons using Bonferroni adjustment for multiple comparisons were performed to locate where significant differences occurred between pairs of means if appropriate.

#### 2.6.3. ICF Blocks

The possible differences in test MEP amplitudes, condition-test MEP amplitudes, and ICF values over the time course of the ICF blocks were analyzed by 3 separate 4 *ICF Block* (ICF_4, ICF_6, ICF_8, ICF_10) × 3 *Epoch* (1,2,3) within-subjects ANOVAs. Post hoc comparisons using Bonferroni adjustment for multiple comparisons were performed to locate where significant differences occurred between pairs of means if appropriate.

The significance level for all statistical tests was set to *p* < 0.05, except when modified by Bonferroni corrections. All data are expressed as means ± standard error in the figures and mean ± standard deviation within the text.

## 3. Results

The group average RMT and 1 mV stimulation intensity (% MSO) were 48.3 ± 7.2 and 55.3 ± 10.1, respectively.

### 3.1. MVCs

The paired *t*-test indicated that there was no significant difference (*p* = 0.06) between the pre-MVC (40.4 ± 13.2 N) and post-MVC force (43.7 ± 13.4 N). Similarly, another paired *t*-test indicated that there was no significant difference (*p* = 0.441) between the pre-MVC EMG (0.89 ± 0.2 mV) and post-MVC EMG (0.84 ± 0.2 mV).

### 3.2. Control Blocks

For MEP amplitudes in the control blocks, the 2 *Control Block* (1 mV_4, 1 mV_10) × 3 *Epoch* (1, 2, 3) within-subjects ANOVA revealed that the main effect for *Control Block* (*p* = 0.721), main effect for *Epoch* (*p* = 0.610), and *Control Block* × 3 *Epoch* interaction (*p* = 0.480) were all non-statistically significant (Figure 2A,B). MEP amplitudes as a function of trial number for the control blocks are depicted for illustration in Figure 3A,B.

### 3.3. ICF Blocks

For test MEP amplitude, the 4 *ICF Block* (TEST_4, TEST_6, TEST_8, TEST_10) × 3 *Epoch* (1,2,3) within-subjects ANOVA revealed that the main effect for *ICF Block* (*p* = 0.893) and main effect for *Epoch* (*p* = 0.976) were both non-statistically significant (Figure 4A and Figure 5A). However, there was a significant *ICF Block* × 3 *Epoch* interaction (*p* = 0.017). Nonetheless, post-hoc analysis of the interaction using Bonferroni adjustment for multiple comparisons indicated that all of the differences between pairs of means were non-significant (all *p*-values > 0.137).

For condition-test MEP amplitude, the 4 *ICF Block* (C-T_4, C-T _6, C-T _8, C-T _10) × 3 *Epoch* (1,2,3) within-subjects ANOVA revealed that the main effect for *ICF Block* (*p* = 0.688), main effect for *Epoch* (*p* = 0.593), and *ICF Block* × 3 *Epoch* interaction (*p* = 0.635) were all non-statistically significant (Figure 4B and Figure 5B). Test MEP and condition-test MEP amplitudes as a function of trial number for the ICF blocks for the four ITIs are depicted for illustration in Figure 6A–D.

**Figure 7:**
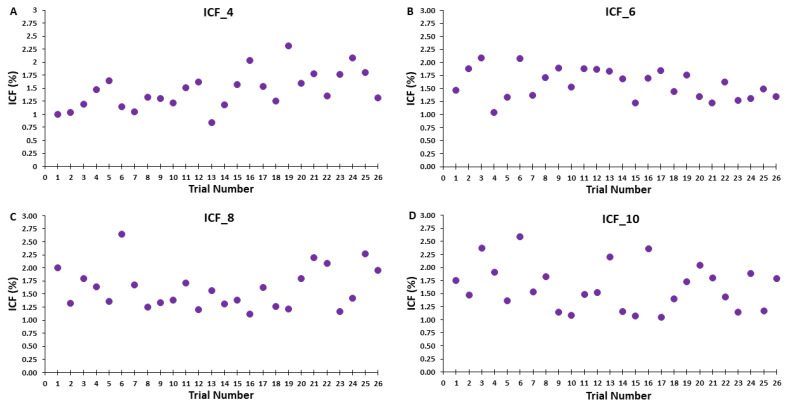
ICF as a function of trial number for the ICF blocks for the four ITIs are depicted for illustration (**A**–**D**). Each data point represents the average ICF values of all 20 subjects for a given trial.

For ICF values, the 4 *ICF Block* (ICF_4, ICF_6, ICF_8, ICF_10) × 3 *Epoch* (1,2,3) within-subjects ANOVA revealed that the main effect for *ICF Block* (*p* = 0.397) and main effect for *Epoch* (*p* = 0.534) were both non-statistically significant (Figure 4C). However, there was a significant *ICF Block* × 3 *Epoch* interaction (*p* = 0.04). Post-hoc analysis of the interaction using Bonferroni adjustment for multiple comparisons indicated that ICF_4 was less than ICF_10 in Epoch 1 (*p* = 0.015; Figure 4C). ICF as a function of trial number for the ICF blocks for the four ITIs are depicted for illustration in Figure 7A–D.

## 4. Discussion

The primary purpose was to examine the influence of different ITIs on ICF measurements at rest, whereas the secondary purpose was to examine the influence of different ITIs on single-pulse MEP measurements at rest. The study produced two sets of main findings: (1) single-pulse MEPs elicited in the short 4-second ITI condition (1 mV_4) and the long 10-second ITI condition (1 mV_10) did not significantly change over the time course of the trial blocks and had similar average amplitudes; (2) ICF values were similar for the four ITIs and did not significantly change over the course of time for any of the ICF blocks. However, the details of the overall ICF results were somewhat nuanced due to a random, non-physiological covariation of data. Taken together, the current findings indicate that ITIs of 4, 6, 8, and 10 s provide nearly identical single-pulse MEP and ICF values.

### 4.1. Effects of ITI on MEP Amplitude in the Control Blocks

MEP amplitude is one of the most common and useful measurements in human motor control studies and provides a global measure of net corticospinal excitability. However, a number of TMS parameters must be considered to ensure proper interpretation of MEP measurements. The possible relevance of ITI on MEP amplitude has been recognized since the early days of TMS research methodology, at least in specific task conditions [21,47,48]. Accordingly, a little over a decade ago the vast majority of an international panel of TMS experts stated that ITI was important to control and should always be reported in TMS studies [15], although there was little if any direct systematic research available on the topic at that time. Despite these recommendations, common observation suggests that very few single-pulse TMS studies report ITI when describing the TMS parameters selected in the research design. This assertion is supported by a systematic review that focused on the related topic of single-pulse TMS reliability measurement, which was reported in an analysis of 16 included studies that only 1 reported the ITI utilized [49]. Therefore, it appears that ITI has been underreported and underappreciated relative to other TMS stimulation parameters with no consensus on the topic as a wide range of constant ITIs and methods of varying the ITI within TMS trial blocks have been used in the literature.

The current study investigated the influence of different ITIs on single-pulse MEPs at rest in 2 control blocks using 4 and 10-second ITIs (1 mV_4 and 1 mV_10 conditions). Although the primary focus of the study was the examination of the effect of ITI on ICF, these blocks served as control measurements for the ICF blocks, especially for the test MEPs elicited in an intermingled fashion with the condition-test MEPs. In addition, the control blocks allowed for comparisons with prior single-pulse TMS studies involving ITI [27,29,30,32,34] in an attempt to confirm or extend previous findings. These studies collectively found that MEP amplitudes were generally decreased at ITIs of 5 s and below compared to longer ITIs. This effect was most prominent in approximately the first 10 trials of a block [27,29,30]. Thus, these results suggest that this phenomenon could lead to inaccurate quantification of the average MEP amplitude over a typical block of MEPs collected in most studies if an ITI of 5 and below is used.

Based on the most relevant previous studies, it was originally hypothesized that MEP amplitude would be lower for the 1 mV_4 block compared to the 1 mV_10 block. This could result from either the initial MEP trials being depressed, a serial reduction in MEP amplitude over the entire block, or a combination of both factors in the 1 mV_4 block compared to the 1 mV_10 block. However, the major findings obtained in the control blocks were contrary to the original hypothesis. First, the first 8 trials that comprised Epoch 1 were not significantly different than the subsequent Epochs 2–3 which contained 8 and 9 trials each in either control block (Figure 2A). Second, MEP amplitudes did not exhibit a serial decrement over the course of either of the control blocks. Third, the overall average MEP amplitudes (25 trials) reflected the previous two findings and were not significantly different between the 1 mV_4 block and the 1 mV_10 block (Figure 2A,B). Fourth, there was no indication of the first trial being substantially different compared to the average MEPs in the same trial block and relative to the normal inherent variability of all of these trials. Fifth, the previous findings and observations are supported by visual inspection of the group average MEP amplitudes plotted as a function of trial number (Figure 3A,B). In summary, single-pulse MEP amplitudes did not display time-varying characteristics within either of the control blocks of 25 trials but rather fluctuated about the average value observed over the entirety of the blocks.

The findings appear to be inconsistent with the results of the majority of previous single-pulse TMS studies. However, many of these dissimilarities are likely due to methodological differences, although a few discrepancies are difficult to resolve. For example, one study [27] found that MEP amplitudes obtained while measuring recruitment curves at rest were significantly lower with an ITI of 5 s compared to an ITI of 20 s. However, recruitment curve construction involves the application of a wide range of stimulus intensities versus constant stimulation intensities used here and in other ITI studies. Furthermore, only 5 MEP trials per condition were averaged in that study, which is common for recruitment curves, but well below the approximate 25 MEPs recommended in most experimental circumstances [45]. Another study reported that MEPs evoked at a constant stimulation intensity were significantly lower at short ITIs (1, 2, 3, and 5 s) compared with a long ITI of 10 s. This was mainly evident in the first 1–10 MEPs of a 30-trial block, which contrasts with the current control block results. However, this study had a number of differences relative to the present study, three of which could be considered major: (1) the sample size consisted of only 8 participants. This is important as the sample size is a large contributor to the estimator error for MEP amplitude quantification [45]; (2) a TMS device that delivered biphasic pulses was used, which can give different results compared with the most common single and paired-pulse TMS devices that give monophasic pulses [50]; and (3) participants watched television (TV) during the experiment as opposed to concentrating on a relatively constant experimental screen where EMG feedback was given. Since MEPs are modulated during the up and down states of neuronal oscillations as measured by EEG and by changes in attention [5], it is uncertain what differential effect TV viewing could have had on results. In another study, Matilainen et al. (2022) found that MEPs were suppressed at a 2-second ITI compared to 5 and 10-second ITIs, although this study also had a low sample size of 9 participants. [32]. In addition, Schmidt et al., (2009) [33] employed a 3-second ITI and observed a transient initial state (~first 20 trials) of MEP amplitudes that differed from subsequent trials, consistent with the other previous studies. However, this study did not compare the 3-second ITI to longer ITIs. In contrast, another report clearly indicated that MEP amplitudes were lower using a 4-second versus a 10-second ITI [34]. These findings were supported by a second study by the same research group that found lower MEP amplitudes at an ITI of 5 seconds compared to 10, 15, and 20-second ITIs. Since these studies used methodology very similar to the current control blocks, the reasons for the different findings are unknown and difficult to reconcile.

In contrast, the present findings are consistent with a few other studies that involved ITI examination as well as physiological studies that provide evidence of why relatively short ITIs are unlikely to cause serial decrements in MEP amplitude. In a classic repetitive TMS study [26], Pascual-Leone and colleagues found that an ITI of 1 second did not influence subsequent MEP amplitudes, and ITIs lower than 1 second were needed to induce time-dependent effects. These direct results are supported by indirect physiological results that demonstrated that a single TMS pulse given to M1 increased cortico-muscular coherence for only 300–800 milliseconds before values returned to baseline [28]. Furthermore, experiments that have evoked single-pulse MEPs in FDI and simultaneously recorded the sequence of descending volleys (direct wave and indirect waves) via an electrode in the cervical epidural space [51,52] have shown that the latest of these waves reach the spinal cord in 10 milliseconds. Thus, it has been argued that single MEPs elicited in hand muscles should theoretically not be influenced by a suprathreshold test MEP stimulus after this time period [53], which is much shorter than the ITIs of 1–5 s in the aforementioned studies. However, this would not necessarily preclude that a series of successive MEPs (e.g., 5–10) at short ITIs of 1–5 s could result in an initial transient suppression of MEP amplitudes. In summary, research is mixed on the impact of short ITIs on MEP amplitude with the balance of studies being in contrast to the current findings, although differences in methodology and interindividual differences in the response to single-pulse TMS across participants in these studies may explain some of these discrepancies.

### 4.2. Effects of ITI on Test MEPs. Condition-Test MEPs, and ICF

The application of a subthreshold conditioning TMS pulse followed by a suprathreshold TMS test pulse at ISIs of between 6–25 milliseconds through the same TMS coil leads to the facilitation of the condition-test MEP amplitudes compared to single-pulse test MEP amplitudes. This phenomenon is termed ICF [6,9,10] and is one of the most common paired-pulse TMS measures of intracortical excitability. The present study was the first to investigate the influence of different ITIs on the quantification of ICF. Based on the limited available data on the influence of ITI on single-pulse TMS measurements, it was originally hypothesized that ICF would be lower in the ICF_4 block compared with the ICF_6, ICF_8, and ICF_10 blocks. This could be due to either the initial ICF trials being depressed, a serial reduction in ICF values over the entire block, or a combination of both factors in the ICF_4 block compared to the longer ITI ICF blocks. Therefore, this could cause the overall average of the ICF_4 block to be lower compared to longer ITIs, which would suggest that ICF measured in previous studies at relatively short ITIs could have yielded inaccurate results. Taken together, the aforementioned hypotheses were not confirmed as ICF values were similar for the four ITIs and did not significantly change over the course of time for any of the ICF blocks. However, the details of the overall results were somewhat nuanced due to a random, non-physiological-based covariation of data as described below.

#### 4.2.1. Effects of ITI on Test MEP Trials Alone

A total of 26 test MEP trials were semi-randomly intermingled with 26 condition-test MEP trials, divided into time epochs, and collectively used to calculate ICF. The test MEP trials were also analyzed alone to determine their specific contribution to ICF. The first 8 test MEP trials that comprised Epoch 1 of the ICF_4 block were technically not statistically different compared with the test MEP trials of Epochs 2 and 3 of ICF_4 and across all the epochs of the other three ICF blocks (ICF_6, ICF_8, and ICF_10). Although there was an *ICF Block* × *Epoch* interaction (Figure 4A), Bonferroni’s post-hoc analyses failed statistical significance (*p* = 0.137) between the ICF_4 and ICF_10 mean pairs in Epoch 1. Based on previous single-pulse ITI studies and this result, one would immediately think that this difference was due to low test MEPs in ICF_4 due to suppression at the short ITI. Crucially, the opposite was true as test MEP amplitudes in ICF_4 were actually much larger than in ICF_10. Thus, there are likely two interrelated reasons for this apparent difference between the two mean pairs: (1) random covariation between the two pairs of means due to the inherent high variability in MEP measurements; and (2) the fact that each epoch only consisted of the average of 8 MEPs makes random occurrences more likely compared to averages across greater numbers of trials (e.g., a whole block). These relationships are clearly reflected in the equations of an extensive review that showed that MEP amplitude estimation error depends on the number of MEP trials and the MEP variability [45]. This topic is covered below when the influence of the test MEP amplitudes in these two epochs on ICF values is discussed.

There was also no evidence of a serial reduction in MEP amplitude over any of the epochs of test MEP trials in any of the ICF blocks (Figure 4A). Test MEP amplitudes did appear to decline slightly over the time course of the ICF_4 trial block, but this small decrease did not reach statistical significance (Figure 5A and Figure 6A). The other ICF blocks displayed even smaller, non-significant serial increases or decreases in test MEP amplitudes over their time courses Figure 5A and Figure 6B–D). Accordingly, the overall average MEP amplitudes (25 trials) reflected the previous findings and were not significantly different between the four ICF blocks (Figure 4A and Figure 5A). These statistical results are supported by visual inspection of the group average test MEP amplitudes plotted as a function of trial number (Figure 6A–D). In summary, test MEP amplitudes alone displayed no differences across epochs or between ICF blocks. The possible exception was a lower technically non-significant MEP amplitude in Epoch 1 for the ICF_4 compared with the ICF_10 block; however, this was likely due to a random covariation of MEP amplitudes between the two conditions in Epoch 1.

#### 4.2.2. Effects of ITI on Condition-Test MEP Trials Alone

The results of the analysis of the condition-test MEP trials alone were more straightforward compared to the test MEP trial results. Overall, the general findings were similar to those attained in the control blocks. The condition-test MEP amplitudes did not display time-varying characteristics over any of the epochs that comprised a total of 26 condition trials in any of the four ICF blocks (Figure 4B). Thus, condition-test MEP amplitudes fluctuated about the average value observed over the entirety of the ICF blocks (Figure 5B). These statistical findings are supported by visual inspection of the group average condition-test MEP amplitudes plotted as a function of trial number (Figure 6A–D). In summary, condition-test MEP amplitudes neither displayed significant time-varying behavior nor different overall average values across any of the four ICF blocks. Thus, these findings would indicate that the condition-test trials alone should provide only small contributions to any differences observed in the quantification of ICF.

#### 4.2.3. Effects of ITI on ICF

**Figure 8:**
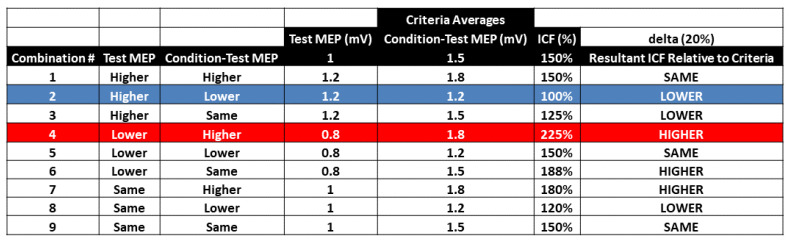
Theoretical mathematical example using created round numbers to illustrate the general concept of how random covariation between the 2 elements of ICF (test MEP, condition-test MEP) for 2 separate epochs (blue and red rows) could influence ICF values and therefore the paired *t*-test between the ICF values for the 2 epochs. This analysis is best explained through the six following steps: (1) ICF is calculated as the ratio of the condition-test MEP amplitude to the test MEP amplitude and then expressed as a percentage; (2) this means that for the ICF value of a specified epoch (blue or red rows) to be higher or lower relative to the average ICF value of other epochs (black row criteria average values, a few mathematical conditions must be met; (3) relative to the average condition-test/test MEP = ICF value (150%), there are 3 possible outcomes (higher, lower, the same) for each of the 2 ICF inputs (test MEP, condition-test MEP). Thus, there are 3 (test MEP) × 3 (condition-test MEP) = 9 combinations that can uniquely influence the value of ICF; (4) 3 leads to a lower ICF vs. the average, 3 leads to a higher ICF vs. the average, and 3 lead to the same ICF vs. the average. Note criteria average test MEP and condition-test MEP absolute values (columns 4–5 from left) were multiplied by 20% (delta 20%) to give a lower or higher change; (4) Epoch 1 of the ICF_4 condition (blue) had a higher test MEP and a lower condition-test MEP than average and therefore lower ICF than average; (5) Epoch 1 of the ICF_10 condition had a lower test MEP and a higher condition-test MEP than average and therefore higher ICF than average; and (6) the low ICF in ICF_4 and high ICF in ICF_10 combined to cause the significant difference in ICF in Epoch 1 between ICF_4 and ICF_10.

All ICF blocks involved 52 TMS trials that included 26 test MEP trials and 26 condition-test MEP trials that were semi-randomly intermingled, divided into time epochs, and collectively used to calculate ICF. Similar to the analysis of the test MEP trial results alone, there was an *ICF Block* × *Epoch* interaction (Figure 4C). However, in this case, Bonferroni’s post-hoc analyses were statistically significant and indicated that ICF was lower in the ICF_4 condition compared with the ICF_10 condition in Epoch 1 (*p* = 0.015). Based on previous single-pulse ITI studies and this result, one would immediately think that this difference could be due to the suppression of test MEPs in ICF_4 or some related problem due to the influence of the short ITI of 4 s. However, a detailed analysis of the test MEP and condition-test MEP amplitudes that caused this difference in ICF values indicated random covariation in a similar manner to that described above in the test MEP results (See Figure 8 for a description and theoretical mathematical example using created round numbers). In summary, the seemingly random covariation of the 4 elements is likely responsible for the difference between the 2 elements of ICF in ICF_4 and ICF_10.

There was also no evidence of a serial reduction in ICF over the remaining time course of the ICF_4 block. In fact, ICF actually increased in Epoch 2 and especially in Epoch 3 in ICF_4 (Figure 4C). The ICF blocks with the longer ITIs also did not display any significant time-varying behavior (Figure 4C). As a result, the overall average ICF values reflected the previous findings and were not significantly different between the four ICF blocks (Figure 4C and Figure 5C). These statistical findings are supported by visual inspection of the group average test MEP and condition-test MEP amplitudes plotted as a function of trial number (Figure 6A–D). Finally, a visual inspection of the resulting ICF values plotted as a function of trial number (Figure 7A–D) shows that ICF randomly fluctuated around the average value for all four ITIs utilized in the ICF blocks. In conclusion, ICF displayed no differences across epochs or between ICF blocks, with the exception of a transient significantly lower ICF value in Epoch 1 for the ICF_4 compared with the ICF_10 block. Notably, this difference quickly disappeared by Epoch 2 between these two conditions. As explained above, this exception was likely due to random covariation of the test MEP and condition-test MEP amplitudes in Epoch 1 for both the ICF_4 and ICF_10 conditions (Figure 4 and Figure 8).

### 4.3. Overall Interpretation of the Combined Control Blocks and ICF Blocks Results

The majority of the current findings were relatively clear. There were negligible, non-significant effects of ITI on the overall single-pulse MEP amplitudes in the control blocks. Similarly, there were negligible, non-significant effects of ITI on single-pulse test MEP and paired-pulse condition-test MEP amplitudes and resulting ICF values. The notable exception was the interrelated outcomes of the test MEP trials and overall ICF values in Epoch 1 of the ICF_4 and ICF_10 blocks. Collectively, several lines of reasoning argue against this one statistical difference being due to an inherent physiological process: (1) the single-pulse MEP amplitudes in both control blocks (1 mV_4 and 1 mV_10) were similar and did not show time-varying characteristics; (2) the test MEP amplitude results in the ICF blocks followed this same pattern. The one exception was the differences in Epoch 1 between ICF_4 and ICF_10. Most importantly, ICF_4 actually had a higher MEP amplitude than ICF_10 in Epoch 1, which would be the opposite finding expected if a physiological mechanism had caused a transient initial suppression of MEP amplitude at the short ITI. Thus, aforementioned exception was almost certainly due to random covariation and the small number of MEP trials per epoch; (3) the condition-test MEP amplitudes were similar across epochs in all four ICF blocks; and (4) ICF also showed no differences across epochs or between ICF blocks, with the exception of a transient significantly lower ICF value in Epoch 1 for the ICF_4 compared with the ICF_10 block. This was also almost certainly a random covariation of the 4 elements (test MEPs and condition-test MEPs in ICF_4 and ICF_10) and not due to physiological suppression of MEPs in the short ITI condition (ICF_4).

### 4.4. Possible Impact of Methodological Issues on the Results

Methodological details are important in TMS studies as a variety of different experimental and biological factors can influence MEP amplitude and likely collectively lead to the well-known high variability of MEPs. Therefore, numerous experimental controls and the most common and standard methods were employed to minimize any confounding influences. The study utilized almost all of the components of methodological quality listed in recent TMS review articles [5,15,49] and similar methods to many of the existing single-pulse ITI studies [27,29,30,32,33,34]. Other notable aspects of the study were the that the participants were young adults (equal numbers of men and women) in a tight age range and strongly right-handed. Finally, pre and post-MVCs were performed and provided confirmation that the ability to voluntarily activate the FDI muscle and by extension factors such as alertness or arousal [5] that could influence MEPs had not substantially declined over the course of the experiment due to central fatigue.

The methodological issue most relevant to the current study was the relatively common practice of excluding the first 1 or first 3–5 MEPs of a trial block from analysis. It can be clearly seen from the results and visual inspection of the figures that such practices are largely unwarranted for ITIs of 4 s and above. This practice originates from the results of two early TMS studies. Flament et al., (1993) [21] reported that the first MEP trial of a block was commonly deleted from analysis due to the tendency for this trial to be larger than all subsequent responses. Since the ISI varied between 3.5 and 7 s in this study, this would seem to imply that ISIs in this range could lead to a small short-term reduction in MEP amplitude beginning after the first trial. Unfortunately, these authors did not provide any data to substantiate deleting the first MEP trial and it seems that this practice was adopted based on subjective observations. In another study, Brasil-Neto et al., (1994) [47] observed a progressive decrement in the first 4 MEPs of a series. However, this was after a fatiguing contraction and therefore not relevant to resting conditions, despite its implied relevance when this topic is mentioned [33]. Finally, an extensive study on MEP reliability found no advantage to deleting the first 3–5 trials of a block of MEPS [54].

### 4.5. Implications and Practical Application of the Findings for ICF Studies

The findings have a least five implications for the practical execution of studies involving both single-pulse TMS and especially ICF measurement: (1) there are no strong reasons to discard the first 3–5 MEP trials of a block for both single-pulse TMS and ICF measurement, especially when ITIs of over 5 s are used; (2) it is probably best practice to be conservative and not combine short ITIs (less than 5 s) and longer ITIs in the same trial block if using randomized ITIs for reasons such as to reduce participant anticipation; (3) based on our pilot work with ITIs over 10 s and the experience with 10 second ITIs in the current study, ITIs above 10 s are not comfortable for the investigators or the participants and are too time consuming in most experimental situations. Most importantly, a prior single-pulse TMS study found no further benefits in MEP measurements for ITIs above 10 s [30]; (4) the common practice of setting the test MEP stimulus intensity to 1 to 1.5 mV seems to allow for relatively accurate MEP measurement for any ITI between 4–10 s. Similarly, previous studies that have used stimulation intensities of 110–120% of RMT support this practice as these result in 1 to 1.5 mV MEPs for most participants [45]; and (5) based on previous studies and the current results, the major point researchers should keep in mind is that there should be no difference in ICF values between ITIs ranging from 4–10 s. Thus, investigators can choose a relatively short ITI if it is more convenient in a given study or a longer one to be conservative and it should not influence the final ICF results. Our overall general recommendation would be to utilize 4–6 second ITIs for both single-pulse TMS and ICF measurements to optimize the trade-off between investigator and participant comfort as well as experimental time efficiency. This would be even more important in clinical studies where patients may be less able to tolerate or maintain concentration in long experiments compared to healthy young adults. Accordingly, we believe the equations and explanations in an extensive review [45] offer an excellent basis for researchers to consider the trade-offs between the number of participants, the number of MEP trials per TMS block, the expected MEP variability, the time available and the number of total TMS blocks needed to answer the research question, and the estimation error deemed acceptable by the investigators.

### 4.6. Limitations

The study had several limitations that should be acknowledged: (1) ITIs of less than four seconds were not tested in either the single-pulse control blocks or ICF blocks, although this was not possible in the ICF blocks due to technical limitations (see Methods). Nonetheless, based on the combined results of several single-pulse studies [27,29,30,32,33,34] any ITI below 4 s should have been sufficient to see ITI-related differences if they existed; (2) single-pulse MEPs or ICF were not tested during active muscle contraction conditions. However, it would be highly unlikely that there would be any effect of ITI on MEP amplitude in these conditions based on previous studies [27,32] and the fact that muscle activation at a set background level provides a much more constant state of corticospinal excitability compared to rest; (3) experimental conditions were not included that varied the ITI trial randomly (within a certain range) as some studies have completed (see Introduction). On the other hand, it is highly doubtful in light of the current results and previous studies [27,29,30,32,33,34] that such an approach would lead to different results, but could be directly tested in future studies as a non-trivial number of TMS studies utilize random ITIs; (4) only ICF was tested and other paired-pulse TMS measures were not investigated. It is possible that other intracortical pathways, especially perhaps inhibitory ones such as SICI, that are mediated by different neurotransmitter and receptor systems, could yield different results. Accordingly, this possibility will have to be investigated in a future study; (5) it could be argued that the sample size of the current study was somewhat small. However, the sample size of 20 was substantially larger than all of the most relevant single-pulse ITI studies (range 8–17; average 12.5 participants) [27,29,30,32,33,34]. The sample sizes typically used in most neuroscience studies could almost always be viewed as a limitation [55,56]; and (6) only one combination of the possible parameters for stimulation (e.g., stimulation intensities of the conditioning and test stimuli, different ISIs) was employed. Although this study used the most common and optimal set of parameters to evoke ICF [11], it can’t be ruled out that different results could emerge. There is evidence that some methods of eliciting ICF could be dependent on different intracortical neuronal populations [12] so further studies could be warranted.

## 5. Conclusions

In summary, the primary findings of the study were that ICF values were similar for all four ITIs (4, 6, 8, 10 s) and did not significantly change over the course of time for any of the ICF blocks. The secondary findings were that single-pulse MEP amplitudes were similar for the 4 and 10-second ITIs and did not significantly change over the time course of the trial blocks. Based on these results, it is recommended that ITIs of 4–6 s be utilized for ICF quantification in TMS studies to optimize participant comfort and experiment time efficiency.

## Data Availability

The data presented in the study are available on request from the corresponding author.
